# Cognitive and neuroimaging markers for preclinical vascular cognitive impairment

**DOI:** 10.1016/j.cccb.2021.100029

**Published:** 2021-10-05

**Authors:** Ellen Lowry, Vaisakh Puthusseryppady, Ann-Kathrin Johnen, Louis Renoult, Michael Hornberger

**Affiliations:** a School of Psychology, University of East Anglia, Norwich NR4 7TJ, United Kingdom; b Norwich Medical School, University of East Anglia, Norwich NR4 7TJ, United Kingdom

**Keywords:** Vascular cognitive impairment, Vascular dementia, Cerebrovascular change, Preclinical dementia, Cardiovascular risk, Cognitive decline in midlife, AD, Alzheimer's disease, VCI, vascular cognitive impairment, DTI, diffusion tensor imaging, APOE-ε4, Apolipoprotein E Allele 4, MCI, mild cognitive impairment, DMN, default mode network, ERPs, event related potentials

## Abstract

Detection of incipient cognitive impairment and dementia pathophysiology is critical to identify preclinical populations and target potentially disease modifying interventions towards them. There are currently concerted efforts for such detection for Alzheimer's disease (AD). By contrast, the examination of cognitive markers and their relationship to biomarkers for Vascular Cognitive Impairment (VCI) is far less established, despite VCI being highly prevalent and often concomitantly presenting with AD. Critically, vascular risk factors are currently associated with the most viable treatment options via pharmacological and non-pharmacological intervention, hence early identification of vascular factors have important implications for modifying dementia disease trajectories. The aim of this review is to examine the current evidence of cognitive marker correlates to VCI pathology. We begin by examining midlife risk factors that predict VCI. Next, discuss preclinical cognitive hallmarks of VCI informed by insights from neuropsychological assessment, network connectivity and ERP/EEG experimental findings. Finally, we discuss limitations of current cognitive assessments and the need for future cognitive test development to inform diagnostic assessment. As well as, intervention outcome measures for preclinical VCI. In turn, these tests will inform earlier detection of vascular changes and allow implementation of disease intervention approaches.

## Highlights

• Explores the cognitive correlates of midlife risk factors of pre-clinical vascular cognitive impairment.
• Midlife cardiovascular risks associated with reduces neural integrity in parietal to anterior structures and may identify at-risk individuals.
• Novel experimental investigation testing fronto-parietal structures is required to increase specificity and sensitivity to pre-clinical VCI and inform clinical testing and diagnostic pathways of at-risk individuals.

## 1 Introduction

Vascular Cognitive Impairment (VCI) is an umbrella term for conditions related to disrupted cerebral blood flow to the brain and subsequent cognitive decline. Traditionally, post-stroke dementia was considered the main contributor to VCI, however it is now clear that it only represents a small portion of VCI, with other incipient neurovascular changes (subcortical, multi-infarct and mixed dementias) significantly contributing to VCI [1]. This also dovetails with findings showing that up to 80% of dementia patients show vascular pathology at autopsy [2] [3] and there appears to be a reciprocal relationship between neurovascular and neurodegenerative pathology [4]. Importantly, cognitive decline in VCI can be insidious and evolve over many years without ‘classic’ stroke symptomology. In the next section, we will review current cognitive markers used in VCI before exploring preclinical cognitive measures and their neural correlates.

The aim of this review is to examine the current evidence of cognitive marker correlates to VCI pathology. We outline the methods used for this narrative review and begin by discussing preclinical hallmarks of VCI informed by insights from neuropsychological assessment, network connectivity and ERP/EEG experimental findings used to detect cognitive dysfunction in individuals at-risk of developing VCI. Lastly, we discuss limitations of current cognitive assessments and the need for new cognitive test development to inform diagnostic assessment and intervention outcome measures for preclinical VCI. In these tests will inform earlier detection of vascular changes and allow implementation of existing disease intervention approaches.

## 2 Method

Several procedures were followed to ensure a comprehensive review of the literature relevant to this narrative review. First, a review of peer-reviewed journals was undertaken using a wide range of key terms including; vascular cognitive impairment, preclinical vascular dementia, midlife cardiovascular risks. Cross-sectional, longitudinal and retrospective studies were reviewed with a focus on experimental studies exploring midlife cognitive and anatomical brain changes, the reference section for each article was searched for additional relevant articles. Databases used included; Neurosynth, Science Direct, PsycARTICLES, PsycINFO, PubMed and Google Scholar. The following publications were searched independently Stroke, Brain, Hypertension, NeuroImage clinical. For the purpose of this review, we define preclinical VCI as a slowing or decline in cognitive or brain function which is associated with the presence of cardiovascular risk factors, before the expression of noticeable symptoms or a clinical criteria is met for VCI. We excluded articles containing diabetes type 1, CADISIL, CAA, post-stroke VCI.

The literature review revealed, the most prevalent risk factors were hypertension, high cholesterol, diabetes type 2 and elevated BMI (see, Table 1.). These were deemed appropriate to investigate as they are a) identifiable with standard clinical assessment, b) modifiable with pharmacological or behavioural intervention and c) highly prevalent within the general population. Each risk factor was then reviewed with a search term postfix of dementia, vascular dementia, vascular cognitive impairment, cognition, cognitive decline, cognitive function, impairment, midlife, middle age, preclinical, prodromal, brain change, visuospatial, executive function, episodic memory, MRI, DMN, EEG, ERP, P300 P3, P3A and connectivity. Based on these findings we were able to establish associated cognitive functions affected in midlife and track the later life outcomes. The cognitive domains featured in the following sections (3.1 - 3.3) were established after this review of the literature which showed repeated deficits in these areas.

**Table 1:** Midlife VCI-Risk factors predictive of cognitive impairment.

**Study Type**	**x̅ age at baseline**	Risk Factor	**Follow up (x̅ years)**	**Outcome Measure**	**Domain**	**Summary**	**Reference**
Retrospective cohort study	46	CAIDE risk score sig measures; Cholesterol >25.9 mg/dl, BMI >30KG/M, sBP >140mm Hg, smoking	36	Dementia diagnosis (unspecified)	non-specific	Modifiable risk factors at midlife are predictive of later-life dementia	Exalto *et al*[5].
Epidemiological cohort study	53	Composite; Diabetes 200 mg/dL, sBP ≥140 mm Hg, dBP 90mm Hg, BMI 25–32%, APOE-ε4 carrier, Lower cognitive function	25	MCI or Dementia diagnosis (unspecified)	non-specific	Cardiovascular risk factors and low cognitive function predict MCI and dementia 20yrs later	Knopman *et al*[6].
Epidemiological cohort study	50	CAIDE risk score sig measures; Age >47yrs, Education <10yrs, sBP >140 mmHg, Total cholesterol 6•5 mmol/L, BMI >30KG/M2, Inactivity <30mins p/w, APOE-ε4 carrier Sex: male	21	Diagnosis of dementia (unspecified)	non-specific	Midlife cardiovascular risk predicts dementia 20yrs later	Kivipelto *et al*[7].
Epidemiological cohort study	58	Higher lipid level: total cholesterol (200–239 mg/dL) and triglycerides (200–500 mg/dL) LDL; 130–159 mg/dL and 160–189 mg/dL	20	Z-scores of DWRT, DSST, WFT DSST	General cognition Executive function, processing speed, sustained attention	Midlife elevated lipid level predictive of 20-year decline on cognition. Association of high LDL and triglycerides greater in DWRT with APOE- ε4 Elevated midlife LDL predictive of selective cognitive decline	Power *et al*[8].
Meta-analysis	54	BMI 27.58 kg/m2 sBP 120.26mm Hg dBP 73.27mm Hg Total Cholesterol 214.99 mg/dL, Glucose level 107.21 mg/dL	20	Composite score of; DWR, DSS, and WF	memory recall, processing speed and sustained attention, phonemic fluency	Composite score cardiovascular risk associated with declined cognitive performance over time	Gonzalez *et al*[9].
Retrospective cohort study	43	High Cholesterol ≥240 mg/dl Borderline cholesterol 200–239 mg/dl	17	Dementia diagnosis (AD sig, VaD trend)	non-specific	57% greater risk of AD 26% greater risk of VaD 50% greater of VaD 23% greater risk of AD	Solomon *et al*[10].
Epidemiology cohort study	57	Hypertension sBP >140 mmHg, dBP 90 mm Hg Prehypertension sBP >120mm Hg, dBP ns	20	Z-scores; DWRT, DSST, WFT DSST	General cognition Executive function, processing speed, sustained attention	Midlife hypertension predicts cognitive decline, DSST most sensitive to preclinical stage. Yet, elevated blood pressure at late life was not associated with cognitive decline	Gottesman *et al*[11].
Epidemiology cohort study	25	Hypertension (BP variability)	25	DSST, RAVLT	Processing speed, sustained attention, verbal memory	Long-term BP variability for 25 years beginning in young adulthood was associated with worse psychomotor speed and verbal memory tests in midlife. Stroop test lacked sensitivity	Yano *et al*[12].
Epidemiological cohort study	71	Prediabetes HbA1c level ≥5.8% Diabetes HbA1c level ≥7.1%	9	MMSE	General cognition	Prediabetes and diabetes at 71 were independently associated with accelerated cognitive decline	Marseglia *et al*[13].
Retrospective cohort study	≥50	Diabetes HbA1c level ≥6.5%	8	Diagnosis of dementia (unspecified)	Non-specific	Diabetes at midlife increases the risk of dementia at follow up	Hsu *et al*[14].
Epidemiological cohort study	62	Diabetes HbA1c level ≥6.68%	14	DSST	Executive function, processing speed, sustained attention	DM at midlife affects cog flexibility and visuospatial abilities but not memory. Word List and Mosaic Task lacked sensitivity	Degen *et al*[15].
Epidemiological cohort study	57	Diabetes HbA1c level ≥6.5%	20y	DSST, WFT	Processing speed, executive function, language, verbal fluency	DM at midlife associated with significant cognitive decline over 20 years compared to controls	Rawlings *et al*[16].
Meta-analysis	50	BMI ≥25KG/M2	3–36	AD and VAD diagnosis	non-specific	Overweight in midlife associated with dementia but continuous BMI in late-life was not associated with dementia	Anstey *et al*[17].
Epidemiological cohort study	36–43	BMI ≥25KG/M2 Gains in waist circumference	30	VMT, Letter Search, Simple RT task	Memory, processing speed, reaction time	Longer exposure to elevated BMI and greater waist circumference at midlife associated with lower cognitive function at 60yrs	Masi *et al*[18].

BMI = body mass index, sBP = systolic blood pressure, dPB = diastolic blood pressure, APOE-ε4 = Apolipoprotein E subtype 4, LDL = low-density lipoproteins, HbA1c = hemoglobin A, DWRT = Delayed Word Recall Test, DSST = Digit Symbol Substitution Test, WFT = Word Fluency Test, RAVLT = Rey Auditory Verbal Learning Test, TMT = Trail Making Test, MMSE = Mini Mental State Examination.

## 3 Preclinical VCI

There is a distinct lack of specificity on the diagnostic criteria for cognitive symptoms in VCI. Indeed, cognitive diagnostic tests in Alzheimer's disease (AD) are largely based on episodic memory deficits, due to the initial impact of AD pathophysiology on medial temporal lobe structures. By contrast, lesion sites in VCI are more heterogeneous and hence symptoms can range from virtually none to multiple cognitive functions being affected. It is therefore not surprising that the characterisation of cognitive deficits in VCI has been highly heterogenous to date. Detection of VCI specific changes in preclinical VCI is therefore even less established, despite offering significant treatment potential.

A different approach is to outline which cardiovascular risk factors predict later-life cognitive impairment. Table 1. Clearly denotes that individuals with VCI risk factors in midlife show a cognitive decline overtime. Below, we review current, limited evidence on more domain-specific cognitive measures associated with established VCI risk factors and examine network connectivity and ERP/EEG contributions to explore potential strategies for the early identification of VCI.

### 3.1 Executive function

Cardiovascular risk is consistently associated with performance decline when high executive demands are required from participants, such as attention and processing speed, similar to clinical VCI and VASCOG criteria. For example, hypertension appears to have the biggest impact on executive function, motor speed and attention and this is most pertinent for hypertension at midlife but not at later-life[19]. Cognitive decline linked to hypertension was shown over a 20-year community-based cohort study[12]. The cognitive battery used in this study consisted of the Delayed Word Recall Test (DWRT), Digit Symbol Substitution Test (DSST) and Word Fluency Test (WFT) administered over three time points over 20 years. Results showed that, on composite scores, individuals on a hypertension spectrum had greater decline over the 20 year duration, compared to individuals with healthy blood pressure, and this effect was independent of age, sex, race, education, body mass index, diabetes mellitus, alcohol consumption, smoking status, ApoE-ε4 genotype, and stroke history[11]. The Digit Symbol Substitution Test was the most sensitive measure to detect this decline. Baseline measures of the Digit Symbol Substitution Test at age 75 years have also been shown to predict the onset of more general cognitive decline, mobility and mood when adjusted for the presence of white matter hyperintensities[20]. Although the Digit Symbol Substitution Test is sensitive to the presence of wider cognitive dysfunction, it has low specificity. Indeed slower processing speeds as detected by this test may serve to identify disorders of cognition, mobility and even mood[21].

Other studies have explored the link of hypertension to processing speed, and the underlying neural changes. For example, it has been shown that hypertension amongst middle aged men (high systolic blood pressure >140 mm Hg) predicted lower grey matter volumes in the supplementary motor area, superior frontal gyrus, anterior cingulate cortex, and left middle temporal gyrus[22]. Low grey matter volume in the supplementary motor area also predicted slower completion times of the Trail Making Test (part B) and poorer recall of items from a four-word short-term working memory test, independent of age, total brain tissue volume, educational history, severity of carotid atherosclerosis, and the extent of periventricular and subcortical white matter lesions[22]. Similarly, diffusion tensor imaging (DTI) studies reported that people with hypertension showed significantly reduced integrity of white matter in the bilateral superior longitudinal fasciculus compared to healthy controls[23] impacting on their processing speed – this change being of particular interest given the propensity for injury to white matter tracts in clinically established VCI[24].

In addition to hypertension, older adults with type 2 diabetes have shown increased mean diffusivity, as measured via DTI, reflecting microstructural white matter abnormalities in the superior longitudinal fasciculus, the uncinate fasciculus, the inferior longitudinal fasciculus, and the genu and splenium of the corpus callosum[25]. This was associated with reduced information processing speed (z scores of; Trail Making Test, Stroop Test, Digit Symbol Substitution Test) and worse verbal memory performance (Rey Auditory Verbal Learning Test) compared to age matched controls, independent of age, sex, estimated IQ, total white matter hyperintensity load, and presence of cerebral infarcts. Cognitive deficits in type 2 diabetic individuals have been found to be weaker in younger adults but still detectable[26], although poor glaucomic control seems to increase the severity of cognitive complaints[27]. Furthermore, in middle age individuals with type 2 diabetes, white matter abnormalities have also been detected using DTI[28], despite no changes shown via resting state fMRI connectivity. Indicating more specific white matter alterations without necessarily affecting the functional connectivity of brain regions. Given that individuals with type 2 diabetes demonstrate greater structural abnormalities characteristic of VCI (white matter hyperintensities and lacunar infarcts) compared to healthy controls, it is promising that these changes appear to be associated with identifiable cognitive markers for symptom identification and tracking.

The presence of executive function decline in VCI is also supported by findings from a prospective study examining changes to cerebral blood flow in healthy older adults with the APOE-ε4 allele dementia-risk-gene[29]. Results showed that APOE-ε4 allele carriers had lower cerebral blood flow, and the effects of this on cognitive performance (Trail Making Test and the Hopkins Verbal Learning Test) were made worse by the co-occurrence of hypertension. However, critically, in individuals with clinically significant hypertension, only those with lower cerebral blood flow demonstrated the negative association between APOE-ε4 and executive function on the Trail Making Test (part B), compared to individuals with higher cerebral blood flow. Yet, there was no interaction between hypertension, APOE-ε4 and cerebral blood flow with respect to the memory recall measured by the Hopkins Verbal Learning Test[29], suggesting that hypertension in individuals at risk of dementia with lower cerebral blood flow impairs executive function selectively. This also implies that greater cerebral blood flow may act as a protective factor against cognitive deficits in APOE-ε4 individuals. This is further confirmed by a cluster analysis investigating the implication of hypertension on cognition, mood and mobility in healthy older adults[30]. Results showed an association between hypertension, reduced Trail Making Test (part B) performance, depressive symptoms and slower speed. Whereas, consistent with more recent findings, memory measures (Hopkins Verbal Learning Test; immediate recall, delayed recall and recognition) did not reveal such associations[30]. This therefore illustrates that visuospatial and set-shifting deficits in individuals at high cardiovascular risk can serve as a potential cognitive marker of VCI, but memory tests may be less sensitive.

Finally, individuals with high composite cardiovascular risk scores also show increased activation change in the left parietal cortex, associated with greater executive demand in the Flanker task, compared to low risk persons[31], establishing a connection between aggregated cardiovascular risk and parietal-mediated cognitive deficits. This nicely dovetails with the DTI findings in the superior longitudinal fasciculus and is consistent with the view that VCI cognitive and functional impairment is frequently more fronto-parietal in nature, as opposed to hippocampal-dependant episodic memory problems that are traditionally associated with AD.

### 3.2 Visuospatial function

Besides the prevalent executive function changes, VCI also often presents clinically with visuospatial deficits, this also seems true before the onset of clinical symptoms. For example, findings from a longitudinal cohort study suggests a steeper decline in visuospatial performance with age for individuals diagnosed with diabetes type 2 at midlife, compared to those diagnosed at a later stage[15]. For those individuals with midlife diabetes, decline over time was observed for the digit symbol test and for visuospatial imagery (where participants are required to count the surfaces of three-dimensional geometrical figures), compared to individuals diagnosed at a later stage[15]. Assessments of word fluency, visual search and verbal recall tasks did not reveal any deficits, suggesting a specific effect of long-term type 2 diabetes in domains of visuospatial processing and processing speed, but not memory.

White matter hyperintensity volume in healthy mid-life adults has also been associated with reduced visuospatial abilities, as assessed using the Hooper Visual Organisation Test[32]. Visuospatial memory and organization performance were worse in participants with greater white matter hyperintensity volumes, as compared to participants with low white matter hyperintensity volumes. A similar pattern of performance was observed for visual scanning and motor speed, assessed by the Trail Making Test. In contrast, verbal memory, abstract reasoning and naming showed no significant differences between individuals with low versus high volume white matter hyperintensity.

Further research shows that young adults with a family history of hypertension (first degree relative systolic with blood pressure >140 mmHg before 60 years old) also have different neural activations during a visuospatial n-back task, compared to controls, despite equivalent task performance[33]. Relative to controls, individuals with a family history of hypertension exhibited lower activation to the visuospatial n-back task in the right inferior parietal lobule and the right inferior temporal gyrus, and substantially more deactivation in the posterior cingulate[33], indicating subtle changes in visuospatial mechanisms in healthy individuals with a predisposition to VCI risk-factors.

### 3.3 Episodic memory

Deficits in episodic memory are less often associated with VCI and more apparent in AD. However, there have been some interesting findings from studies with at VCI-risk individuals, suggesting that brain regions involved in episodic memory are also affected by early neurovascular change. In a cross-sectional study, reduced scores in immediate and delayed Emotional Memory and free recall in the California Verbal Learning Test were found in middle aged individuals with type 2 diabetes, compared to healthy controls. In contrast, working memory, sustained attention and verbal fluency were unaffected[34]. DTI analysis showed that white matter microstructural abnormalities were present amongst individuals with diabetes and were predominantly located in the frontal and temporal regions, particularly the left temporal stem[34]. These DTI findings may explain the lower memory performance present amongst diabetics, after accounting for age, metabolic dysregulation and hypertension. This is supported by a further cross-sectional study indicating that older adults with type 2 diabetes had poorer performance on the Rey Complex Figure Test (memory recall condition) and longer completion times in the Stroop test[35]. These results were associated with grey matter loss in the anterior cingulate and medial frontal lobes, and with white matter loss in frontal and temporal regions. Interestingly, longer diabetes duration (≥15yrs) was associated with impairments in visuospatial and inhibition domains, with reduced performance in the Rey Complex Figure Test (copy condition), digit symbol coding and digit search over time[35].

In addition, older adults with greater cardiovascular burden have been shown to have an accelerated decline in episodic memory, working memory, and perceptual speed over time compared to lower risk individuals[36]. Episodic memory was assessed using Word List Memory, Word List Recall, Word List Recognition and immediate and delayed recall of the Wechsler Memory Scale-revised. Working memory was evaluated using the Digit Span and digit ordering tasks. Perceptual speed was tested using the Symbol Digit Modalities Test, Number Comparison, and 2 indices from a modified Stroop Test. MRI analysis revealed that reduced episodic and working memory performance was associated with smaller volumes of the hippocampus, whilst reduced perceptual speed was linked to greater volume of white matter hyperintensities[36]. This is supported by an earlier study examining cognition in mild cognitive impairment (MCI) individuals with hippocampal atrophy compared with MCI individuals with severe white matter hyperintensities. Results show equally impaired episodic memory performance (using an object-colour association task) for both groups but with additional impairment for the MCI individuals with white matter hyperintensities in tests tapping verbal and spatial working memory abilities and attentional control processes[37]. This potentially suggests white matter hyperintensities reflect disruption to the white matter tracts (dorsolateral prefrontal cortex and connected neural circuits), which result in diminished executive control processes critical to working memory, that may in turn impair episodic memory function[37].

This account of insidious pathology in VCI-risk populations indicates that early manifestations of VCI may also span medial temporal and related networks. However, at this stage it is unclear whether these episodic memory impairments are in line with age-related memory decline, concomitant AD or a collateral effect of VCI. Regardless, it emerges that the cognitive changes in preclinical VCI might more affect networks of brain regions, instead of discrete areas, in particular when white matter tracts are affected. In the following section we will review therefore how potentially the network connectivity between brain regions might be affected in preclinical VCI and how this will impact on the cognitive symptomology.

## 4 Network connectivity

### 4.1 fMRI

Disruptions to the default mode network (DMN) is of major interest in preclinical dementia. The DMN consists of a network with three major subdivisions: the ventral medial prefrontal cortex, the dorsal medial prefrontal cortex and the posterior cingulate cortex and adjacent precuneus plus the lateral parietal cortex[38][39] that co-activate when subjects are at rest and de-activate when subjects become engaged in external cognitive tasks. It is thought that DMN dysconnectivity could represent a biomarker for preclinical AD and this may be related to cerebrovascular function[40][41]. Cerebrovascular reactivity was significantly reduced in the posterior cingulate/precuneus and anterior cingulate areas of the DMN for those with hypertension and prehypertension (defined as either systolic blood pressure >= 130 mmHg, diastolic blood pressure >= 85 mmHg or use of blood pressure medication) as compared to healthy controls[41]. Similarly, MCI patients with evidence of cerebrovascular infarcts also showed differing underlying (de)activation patterns compared to MCI individuals free from infarcts, during an n-back task, even though neuropsychological scores were similar[42]. Results indicate impaired deactivation in the precuneus/ posterior cingulate cortex, for those MCI individuals with infarcts. Additionally, greater activation was observed in the anterior cingulate gyrus during differing memory loads compared to MCI patients without infarcts. To suggest a differing working memory load fMRI response between groups and potentially indicating different DMN connectivity for preclinical VCI individuals as compared to early stage AD individuals and would also nicely dovetail with the white matter changes reported in preclinical VCI (though see, [43]).

Frontal networks, including the salience network primarily composed of the anterior insula and the dorsal cingulate cortex, involved in sustained attention and task switching, have also been implicated in dysconnectivity in MCI individuals with elevated mean systolic blood pressure (150 mmHg), compared to healthy aged matched controls[40]. The resting state fMRI study in MCI, healthy age matched controls and young adults showed that the salience network, particularly its dorsal sub-network, modulates the interactions between the DMN and the central executive network in young adults and healthy ageing. This pattern of modulation in the salience network was interrupted in MCI and the degree of disruption was associated with lower overall cognitive scores on the MoCA[40]. Importantly, the authors commented that the effect was particularly pronounced in the MCI individuals due to their elevated blood pressure and more pervasive ‘executive’ impairments assessed by the MoCA, TMT, Stroop interference and verbal frequency. Based on these findings, salience network changes might be indicative of measuring frontally mediated executive impairment and asocial vascular pathophysiology in MCI. This would also dovetail with findings showing that dysfunction to the DMN/ salience networks and associated functional impairment may be predictive biomarkers of later-life VCI[44] [45],.

### 4.2 ERP/EEG

Network dysfunction can not only be measured via fMRI but also via EEG derived measures such as event-related potentials (ERPs) or frequency analyses. This approach has been successfully employed to patients presented with clinical dementia syndromes[46]. The P300 component in particular, has been suggested as a physiological marker of preclinical AD[47]. P300 is thought to be evoked by the rapid detection of environmental changes and is associated with domains of working memory as well as attention and characterized by a positive deflection with a latency between 250 and 500ms[48]. A classic paradigm for evoking the P300 response is the oddball task, where participants have to detect rare stimuli amongst frequently occurring standard stimuli. ‘Oddball’ target stimuli typically evoke a response that is maximal at parietal electrode sites (P3b), whilst novel stimuli evoke a response that is maximal at frontal sites (P3a [49];). These novel and target P300 components can be used to evaluate cognitive decline[47] and evidence suggests that decreased P300 amplitude is associated with reduced language, memory and executive function performance in AD[48]. Additionally, non-clinical older adults with reduced cognitive ability (MoCA score <25) demonstrate reduced P300 amplitude compared to age matched controls and healthy young adults[50]. Reduced P300 latencies have also been observed in MCI and in AD, compared to healthy controls (for a review see, [51]).

Importantly, one study reported that VCI and AD could be differentiated by including novel stimuli in an auditory oddball paradigm[52]. Results showed that the novel P300 amplitude (P3a) was markedly reduced in VCI patients, but that it was preserved in AD and controls[52]. Further, source localisation studies indicate that VCI patients have an impaired parietal-to-frontal and parietal-to-central connectivity during the oddball paradigm (200–300 ms after stimuli onset) compared to controls, showing a weakened outgoing connectivity from parietal regions[53][54]. This response appears different compared to AD groups where a shift of maximum intensity location during the P300 response occurs from the frontal-to-temporal lobes[55]. The P300 component could also be a promising biomarker in preclinical individuals as the ‘oddball’ response in middle age type 2 diabetics[56] and hypertensive older adults[57] includes increased P300 latencies compared to healthy controls. Taken together, the evidence suggests this differing ERP response may reflect dysconnectivity between posterior and anterior structures in at VCI-risk individuals – reminiscent of white matter tract dysfunctions in preclinical and clinical VCI individuals. Below, in Fig. 1. we have provided a schematic illustration of the brain regions implicated at the prodromal stage of VCI and a potential preliminary model of emerging disconnectivity.

**Fig.. 1.**
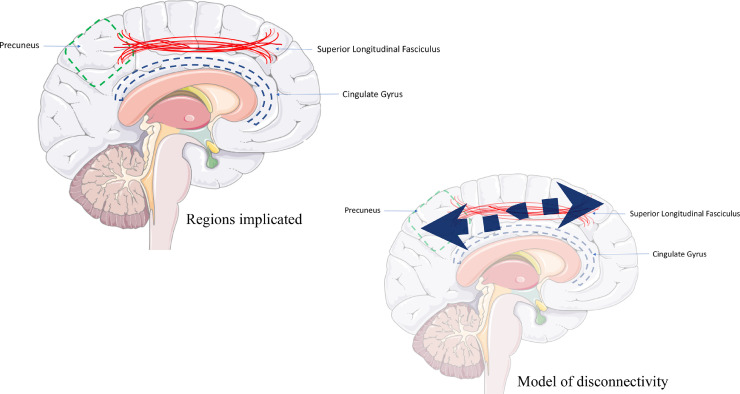
Schematic illustration of brain regions implicated and potential model of disconnectivity emerging between parietal sites and connecting anterior regions. Brain image courtesy of Servier Medical Art https://smart.servier.com/.

## 5 Conclusion and outlook

There is clearly a disconnect between well-established neural markers of VCI and how they relate to cognitive symptomology. This may be explained by the current lack of a gold standard or single domain cognitive measures to detect VCI, either clinically or preclinically. Instead current cognitive measures lack the specificity to isolate VCI pathology and sensitivity to detect its earliest symptoms. This might not be a problem per se, however clinically a functional readout of how symptoms change over time, including cognitive problems, is important for disease prevention, management, and tracking.

Taken together, the reviewed evidence suggests that impaired executive function, visuo-spatial and set-shifting ability along with dysconnectivity between the frontal-parietal networks and subcortical structures (cingulate, insula and precuneus) may predict VCI onset. The VASCOG guidelines[58] highlight that disturbances to frontal and executive processes (slowed information processing, reduced set-shifting ability and poorer working memory) are prominent in VCI. Although this contributes towards identifying the cognitive and biological markers of preclinical VCI, the current clinical measures (MoCA, TMT, DSST…) might not be sensitive or specific enough to detect the earliest VCI changes or disassociate it from incipient AD pathophysiology (see, Table 2. for the neural substrates of typical cognitive symptoms of VCI compared to AD). Instead, more experimental measures informed by preclinical neural and cognitive change need to be explored to fill the gaps in this research area and aid our understanding of biomarker development of this insidious multifaceted disease.

**Table 2:** Typical neural substrates of cognitive symptoms of VCI compared to AD.

	**Frontal**	**Parietal**	**Temporal**
VCI	+++	++	+
AD	+	+	+++

Strength of symptoms, + low; ++ moderate; +++ high.

However, one particularly striking aspect emerging from our review is that despite parietal functions, areas, and connections being commonly affected in VCI, cognitive and neural measures rarely tapped into those. In addition, the superior longitudinal fasciculus (white matter tract connecting parietal, temporal with prefrontal regions) is heavily implicated in underpinning executive function[59] but also likely plays into parietal dysfunction in VCI. Interestingly, this white matter tract is also thought to play a role in spatial processing[60]. As such, investigations into parietal mediated spatial orientation could be a potentially novel and more specific approach towards VCI and may help to discriminate between VCI and AD pathology. In a recent study, we have showed that indeed egocentric (self-referential) spatial orientation has sensitivity and selectivity to VCI and can be used as a measure to differentiate between VCI and AD patients[61]. This is supported by an earlier case study suggesting that egocentric orientation deficits, mediated by fronto-parietal structures, may be a promising marker for the identification of vascular pathology[62]. This coupled with MRI and ERP experimental findings from VCI and at-risk individuals may mark a promising path forward for the development of selective markers focused on connectivity changes across key brain structures.

Although neuroimaging is less accessible for clinical utility, it is clear network approaches should be explored to identify at VCI-risk individuals to help inform more selective cognitive testing. Although, this area of research is in its infancy the present review suggests neuropsychological assessments which tap into structures affected at the preclinical stage of VCI (see, outcome measures listed in Table. 1) are more appropriate for diagnostic and screening purposes compared to more multi-domain assessments. Yet, new and novel methods assessing self-referential spatial navigation seem to have greater selectivity and specificity than more traditional pen and paper tasks (see, [61]).

However, the current review is not without limitation. VCI is highly heterogenous in terms of expression, as well as pathogenesis. Therefore, have not considered the complex interaction of risk factors (for review, see [63]), or how they may contribute towards potential different expressions of preclinical VCI. Nor, have we discussed the latter stages of VCI at its clinical threshold or how specific lesion sites may be incorporated into our preliminary model (see, Fig. 1) of prodromal change. This is due to this review being focused on modifiable mechanisms that contribute to cerebrovascular change with an aim to identify their cognitive fingerprints at an early stage. However, longitudinal tracking of the risk factors, along with cognitive and neuroimaging markers discussed above, may help identify at-risk individuals and a potential ‘sensitive window’ to deduce if/or when symptomology could be slowed or halted with appropriate intervention. We also acknowledge that the body of literature examining cognitive and neuroimaging correlates of risk factors that predate VCI is limited and has highlighted the need for further resources and investigation to gain a broader understanding of the preclinical stage.

Growing evidence indicates that cardiovascular risk factors are associated with increased structural and metabolic change in the brain, worse cognitive performance and increased dementia risk. Yet, research is still lacking in detecting these symptoms in preclinical individuals before presentation of frank VCI. However, from the research discussed throughout this review there appears to be an emergence of cognitive dysfunction and connectivity changes in at VCI-risk individuals and as such, a more subtle and sophisticated approach is required to detect these biological and behavioural markers in their earliest manifestations. Focusing investigation into new and novel screening techniques to detect this dysfunction is key to providing clinical utility in order to identify, intervene and track disease progression as well as personalise treatment pathways.

## Author contributions statement

EL, MH and LR contributed to the conception, designed and the intellectual contribution to the writing of the manuscript. VP and AJ provided intellectual contribution to the manuscript.

## Funding

This work was supported by the School of Psychology at the University of East Anglia.

## Declaration of Competing Interest

All contributing authors have no competing interests.
